# Risks of developing breast and colorectal cancer in association with incomes and geographic locations in Texas: a retrospective cohort study

**DOI:** 10.1186/s12885-016-2324-z

**Published:** 2016-04-26

**Authors:** Zheyu Liu, Kai Zhang, Xianglin L. Du

**Affiliations:** Department of Epidemiology, Human Genetics, and Environmental Sciences, School of Public Health, University of Texas Health Science Center, 1200 Pressler Street, RAS-E631, Houston, TX 77030 USA; Department of Biostatistics, School of Public Health, University of Texas Health Science Center, Houston, TX USA; Department of Epidemiology, Human Genetics, and Environmental Sciences and Center for Health Service Research, School of Public Health, University of Texas Health Science Center, Houston, TX USA

**Keywords:** Cancer incidence, Breast cancer, Colorectal cancer, Income, Geographic variation, Spatial analysis

## Abstract

**Background:**

No study has been conducted to investigate the spatial pattern and association of socioeconomic status (such as income) with breast and colorectal cancer incidence in Texas, United States. This study aimed to determine whether median household income was associated with the risk of developing breast and colorectal cancer in Texas and to identify higher cancer risks by race/ethnicity and geographic areas.

**Methods:**

This was a retrospective cohort study with an ecological component in using aggregated measures at the county level. We identified 243,677 women with breast cancer and 155,534 men and women with colorectal cancer residing in 254 counties in Texas in 1995–2011 from the public-use dataset of Texas Cancer Registry. The denominator population and median household income at the county level was obtained from the U.S. Bureau of the Census. Cancer incidence rates were calculated as number of cases per 100,000 persons and age-adjusted using the 2000 US population data. We used the ArcGIS v10.1 (geographic information system software) to identify multiple clustered counties with high and low cancer incidences in Texas.

**Results:**

Age-adjusted breast cancer incidence rate in the highest median income quintile group was 151.51 cases per 100,000 in 2008–2011 as compared to 98.95 cases per 100,000 in the lowest median income quintile group. The risk of colorectal cancer appeared to decrease with increasing median income in racial/ethnic population. Spatial analysis revealed the significant low breast cancer incidence cluster regions located in southwest US-Mexico border counties in Texas.

**Conclusions:**

This study demonstrated that higher income was associated with an increased risk of breast cancer and a decreased risk of colorectal cancer in Texas. There were geographic variations with cancer incidence clustered in high risk areas in Texas. Future studies may need to explore more factors that might explain income and cancer risk associations and their geographic variations.

**Electronic supplementary material:**

The online version of this article (doi:10.1186/s12885-016-2324-z) contains supplementary material, which is available to authorized users.

## Background

Breast cancer has the highest incidence rate in women and colorectal cancer is the third most common cancer in both men and women in the United States [1–4]. Previous studies had shown that breast cancer is associated with higher socioeconomic status (SES, such as higher income) and colorectal cancer is associated with lower SES in the U.S [5–13]. A study of cancer prevention using data from the California Cancer Registry showed that breast cancer incidence increased substantially with increasing SES [12]. A comprehensive review of SES related to breast and colorectal cancer in 11 registries of Surveillance, Epidemiology, and End Results (SEER) Program showed the similar findings of an increased breast cancer incidence and a decreased colorectal incidence with higher SES [5, 11, 14]. Previous studies have used spatial pattern analysis to identify areas with high breast [15–18] and colorectal [19, 20] cancer incidence associated with socioeconomic factor. A study on the geographic distribution of late stage breast cancer cases has shown that higher breast cancer incidence rates were significantly associated with higher SES level in Florida between 1998 and 2002 [16]. However, no study has been conducted to investigate the spatial pattern and association of SES with breast and colorectal cancer incidence in Texas. Previous studies have suggested using education, income and occupation may represent different aspects of SES and one of these indicators should be used in epidemiologic studies involving SES [21]. Therefore, this study used the Texas Cancer Registry (TCR) database to determine the association of median household income with breast and colorectal cancer incidence rates from 1995 to 2011 in Texas [22]. Furthermore, we conducted cluster analysis to identify the counties with excessive high or low variation of breast and colorectal cancer incidence. The median household income at the county level in Texas was obtained from the U.S. census data [23]. Because individual level SES data were not available, group level SES data were frequently used to examine its association with cancer risk in the U.S. and in Europe [16, 20, 24–26].

Here, we classified counties into five median household income categories by calendar year to examine whether median household income was correlated with the risk of breast and colorectal cancer in Texas [7, 27, 28]. Additionally, geographic maps were utilized to highlight the spatial differences in particular regions with excess disease rate. Moreover, we examined whether the relationship between median household income and the risk of breast and colorectal cancer interacted with race/ethnicity and metro/urban/rural status. The findings from this study of both breast and colorectal cancer can help identify high risk populations and regions with respect to breast and colorectal cancer, which can enhance cancer prevention and control.

## Methods

### Study design and data sources

This was a retrospective cohort study with an ecological component in using aggregated measures at the county level. The Texas Cancer Registry (TCR) granted the permission to access the public-use dataset which was used to identify incident breast and colorectal cancer cases. The TCR is a statewide and population-based cancer registry with gold certification by the North American Association of Central Cancer Registries [22]. The TCR determined to cover at least 95 % statewide cancer cases diagnosed from 1995 to 2011 in Texas. Information on county population estimates, median household income, and population age groups was obtained from the U.S. Census data in 2000 and 2010 without needing permission [23, 29]. County median household income data represented gross income from all sources, including government transfers but excluding non-cash benefits. The Institutional Review Board of the Texas Department of State Health Services and the Committee for the Protection of Human Subjects at the University of Texas Health Science Center granted ethics approval to our study. The informed consent was waived because the study was retrospective in design and from public datasets.

### Measure of median household income

Because individual income information was not available at the TCR dataset, the aggregated median household income at county level was analyzed as SES. County median household income was chosen in this study because the median household income was more homogeneous with respect to SES and more accessible with wide representative of individual income factor [30]. Previous studies have frequently used these county-level socioeconomic indicators (ex. county poverty, and county median household income) to study temporal trends with breast and colorectal cancer incidence rates in U.S [12, 16, 31–34].

Median household income at the county level was obtained from the U.S. Census Bureau [23]. It was calculated by 4 time periods according to calendar year (1995–1999, 2000–2003, 2004–2007, and 2008–2011), and income value in each time period was calculated as mean of incomes in all calendar years in the period. Because median household income at county level was not available in 1996, income in the 1995–1999 period was a mean of incomes in 1995, 1997, 1998 and 1999. Median household incomes in all 254 counties were then classified into quintiles with approximately equal number of counties in each of 5 income categories, ranging from the highest median household income (5^th^ quintile) to the lowest (1^th^ quintile) in Texas.

### Breast and colorectal cancer cases

Incident breast and colorectal cancer cases were identified from TCR data using the following criteria: breast cancer among women and colorectal cancer among both men and women, diagnosed between 1995 and 2011, and no missing records on county at diagnosis. Breast cancer cases were identified using the “Primary Site” variable in TCR, coded as C500-C509 according to “International Classification of Diseases for Oncology, Third Edition (ICD-O-3), and Topography Section” [35]. Colorectal cancer cases were coded as C180-C189, C199, C209, and C260. According to the methods by Wu et al. [36] in counting total colorectal cancer cases, colon included the cecum (C180), appendix (C181), ascending colon (C182), hepatic flexure (C183), transverse colon (C184), splenic flexure (C185), descending colon (C186), sigmoid colon (C187), and large intestine, NOS(C188-C189,C260). The rectum included the rectosigmoid junction (C199) and the rectum-not otherwise specified (C209). In Texas, 243,677 women with breast cancer and 155,534 men and women with colorectal cancer residing in 254 counties from 1995 to 2011 were identified. Those breast and colorectal cancer cases with unknown county record were excluded (*n* = 35). Using TCR dataset, we obtained age, sex, and race/ethnicity for breast and colorectal cancer cases at an individual level [22]. Cases were separated into five age groups and four race/ethnicity categories. Five age groups were defined as <50, 50–59, 60–69, 70–79, and >79 years old. Four race/ethnicity categories were defined as non-Hispanic white, non-Hispanic black, Hispanic, and other. The other category includes Asian, Pacific Islander, American Indians, and unspecified race/ethnicity in TCR dataset. Definition of metro/urban/rural Texas county code were obtained from 2003 version of the U.S. Department of Agriculture (USDA) urban/rural continuum codes (RUCC). The USDA RUCC categorized counties as metropolitan (RUCC 1–3), nonmetropolitan with urban populations (RUCC 4–7), or rural (RUCC8-9) [37].

### Statistical analyses

Spatial analyses have become an important tool used in public health research to identify potential cluster disease regions [15–20]. In this study, we first calculated the adjusted incidence rates at county level for breast and colorectal cancer separately after controlling for age and median household income, and then evaluated whether incidence rate clusters existed using the Getis-Ord G’s statistic tool in ArcGIS 10.1 (ESRI, Redlands, CA) (Additional file 1) [38]. We also generated all maps in the figures and in supplemental materials using the ArcGIS 10.1 software [38].

The denominator of population data used to calculate incidence rates were acquired from the U.S. Census Bureau’s Population Estimates Program [29]. Because age is a strong confounder and failure to use age-adjusted incidence rates in cancer study may lead to an underestimation or overestimation of incidence rates, we presented age-adjusted incidence rates as number of new cases per 100,000 persons which were standardized to the 2000 US population by five age groups and 4-year periods from 1995 to 2011 [12, 39, 40]. One assumption was that the median household income and population size of given counties would not change dramatically in each study period. Other cancer studies supported this assumption and showed no appreciable changes in aggregated median household income measured at the county levels over each study period [41, 42].

Furthermore, cancer incidence rates were stratified by tumor stage for each median household income categories. Tumor stage at diagnosis includes in-situ, localized, regional, distant, and unstaged, which were defined according to the staging manual of National Cancer Institute [35]. The in-situ stage was defined as “the presence of malignant cells within the cell group from which they arose”. Localized stage was defined as “a malignancy limited to the organ of origin; it has spread no farther than the organ in which it started”. Regional stage was defined as “tumor extension beyond the limits of the organ of origin”. Distant stage was defined as “tumor cells that have broken away from the primary tumor, have travelled to other parts of the body, and have begun to grow at the new location”. In this study, we combined category of in situ and localized as early cancer stage, region and distant as late cancer stage. As a result, it allowed for an assessment of whether or not early or late stage breast and colorectal cancer incidence rates were positively associated with median household income factor. Poisson regression model, which is often used to model the rare disease, was chosen to model the number of cases in each county [43]. Poisson regression model with population size specific to demographic groups as an offset variable was used to determine the association between incidence rate ratio (IRR) and median household income, adjusting for age, gender, race/ethnicity, degree of urbanization/population, and all two-way interaction terms (Additional file 1). In order to determine the temporal relationship, incidence rate ratios were calculated separately and adjusted by potential confounders in each time period. The assumptions of the Poisson regression model were examined by linearity, constant variance and independent structure of observations. The examination showed only a minor degree of overdispersion, supporting that the Poisson regression model assumption was acceptable. The SAS 9.3 statistical software (SAS Institute Inc., Cary, NC) was used on all analyses.

## Results

### Trends in breast cancer incidence rates

Table 1 presents the distribution of age-adjusted breast cancer incidence rates stratified by median household income and tumor stage factors in Texas from 1995 to 2011. Overall age-adjusted breast cancer incidence rates were 153.87, 157.58, 142.81, and 141.07 cases per 100,000, respectively by 4 time periods (1995–1999, 2000–2003, 2004–2007, and 2007–2011). Breast cancer incidence increased from 1995 to 2003 and decreased from 2004 to 2011. The increasing breast cancer incidence in 1995–2003 was consistent with the time period when the widespread use of screening program was implemented [44, 45]. Within each time period, there was a significant association between breast cancer incidence and median household income level. For example, breast cancer age-adjusted incidence rate in the highest median income quintile group was 151.51 cases per 100,000 in 2008–2011 as compared to 98.95 cases per 100,000 in the lowest median income quintile group. After the results were stratified by tumor stage (last 2 columns in Table 1), the association between higher income and an increased breast cancer incidence was largely limited to women with early stage breast cancer, while there was no clear pattern of an association between high income and late stage breast cancer incidence.

**Table 1:** Number of women^a^ diagnosed with breast cancer, population estimates^b^, and age-adjusted breast cancer incidence rate^c^ in Texas, 1995–2011, stratified by median household income and tumor stage^d^ factors

Breast cancer: median household income quintiles	Counties	Cases^a^	Population^b^	Crude incidence rate per 100,000	Age-adjusted incidence rate per 100,000^c^	Early stage age-adjusted incidence rate per 100,000^c,d^	Late stage age-adjusted incidence rate per 100,000^c,d^
1995–1999							
Income Low < = $24,561	51	3,611	3,866,891	93.38	112.12	74.62	37.86
$24,561 < Income 2^nd^ < = $27,991	51	4,746	3,799,073	124.93	133.27	88.80	44.36
$27,991 < Income 3^rd^ < = $29,928	51	5,569	3,760,530	148.09	147.20	103.60	43.92
$29,928 < Income 4^th^ < = $33,652	51	14,210	10,583,649	134.26	150.55	106.60	43.93
$33,652 < Income High < = $68,003	50	35,470	27,844,877	127.38	166.25	118.21	47.93
Total 1995–1999	254	63,606	49,855,020	127.58	153.87	108.32	45.56
2000–2003							
Income Low < = $27,903	51	2,967	2,998,469	98.95	114.85	81.19	33.81
$27,903 < Income 2^nd^ < = $30,837	51	4,310	3,575,541	120.54	131.13	89.05	41.98
$30,837 < Income 3^rd^ < = $33,259	51	4,654	3,299,143	141.07	143.66	100.71	43.16
$33,259 < Income 4^th^ < = $37,476	51	7,568	5,025,927	150.58	155.20	111.73	43.65
$37,476 < Income High < = $76,188	50	38,077	28,387,872	134.13	168.50	121.09	47.33
Total 2000–2003	254	57,576	43,286,952	133.01	157.58	112.52	45.05
2004–2007							
Income Low < = $31,024	51	2,901	3,184,129	91.11	104.48	77.26	45.47
$31,024 < Income 2^nd^ < = $34,102	51	4,209	3,846,869	109.41	118.38	82.69	51.54
$34,102 < Income 3^rd^ < = $37,540	51	6,271	4,491,946	139.61	137.83	96.68	52.90
$37,540 < Income 4^th^ < = $42,068	51	6,290	4,600,724	136.72	136.46	96.73	49.81
$42,068 < Income High < = $75,467	50	38,373	30,404,444	126.21	152.32	106.24	47.39
Total 2004–2007	254	58,044	46,528,112	124.75	142.81	100.50	48.52
2008–2011							
Income Low < = $34,647	51	3,117	3,467,854	89.88	98.95	73.97	41.65
$34,647 < Income 2^nd^ < = $38,040	51	5,041	4,502,366	111.96	119.40	87.47	45.37
$38,040 < Income 3^rd^ < = $42,072	51	6,448	4,582,487	140.71	132.31	95.02	49.30
$42,072 < Income 4^th^ < = $48,438	51	17,738	13,333,909	133.03	144.10	104.09	43.68
$48,438 < Income High < = $80,876	50	32,107	24,519,373	130.95	151.51	111.76	41.16
Total 2008–2011	254	64,451	50,405,989	127.86	141.07	103.59	43.08

^a^Cases with unknown county were excluded

^b^Female population estimates were obtained from US Census Bureau's Population Estimates Program

^c^Incidence rate was number of cases per 100,000 population, and was age adjusted to the 2000 US population

^d^Breast cancer early stage includes in situ and localized. Late stage includes regional and distant

**Fig. 1:**
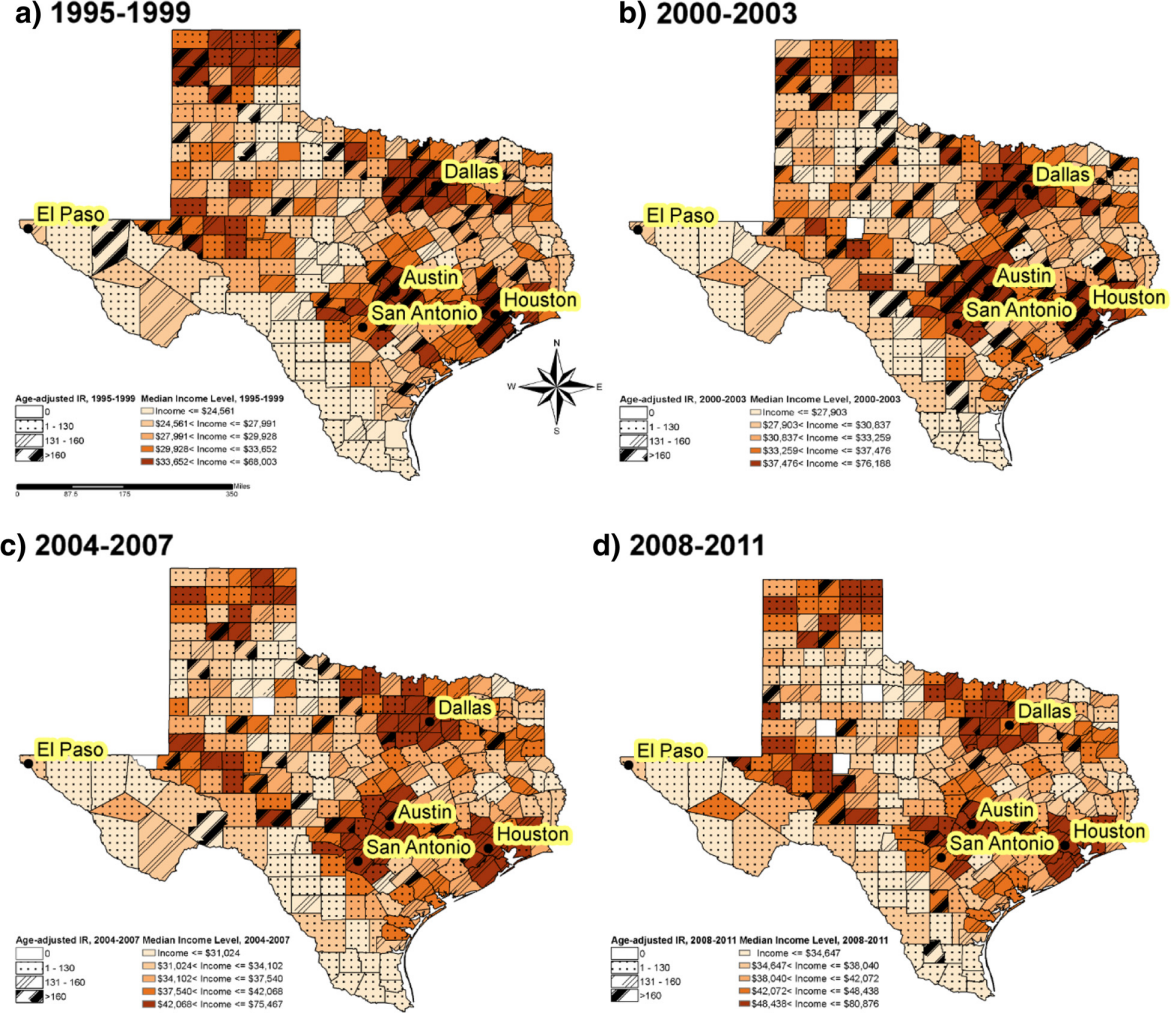
Geographic variations of breast cancer incidence adjusted for age and median household income in Texas, 1995–2011

Figure 1 provides the geographic distribution of age-adjusted breast cancer incidence rates associated with median income at county level, stratified by four time period, (a) 1995–1999, (b) 2000–2003, (c) 2004–2007, and (d) 2008–2011. Counties with higher median income were likely to have higher breast cancer incidence rates. The effect of increasing median income quintiles on the age-adjusted breast cancer incidence rates was most pronounced in 2000–2003. Lowest median income counties were located around US-Mexico border areas, where age-adjusted breast cancer incidence rates were low. Spatial analysis revealed the significant low breast cancer incidence cluster regions located in southwest US-Mexico border counties in every study time period (*P* < 0.001). In other areas of Texas, only a few isolated counties were identified as low cold spot regions. Cold spot maps were provided in supplemental materials (Additional file 1: Figure S1).

### Trends in colorectal cancer incidence rates

Table 2 presents the distribution of age-adjusted colorectal cancer incidence rates stratified by median income from 1995 to 2011. Overall age-adjusted colorectal cancer incidence rates were 59.17, 58.11, 52.49, and 45.76 cases per 100,000, respectively for the 4 time periods (1995–1999, 2000–2003, 2004–2007, and 2007–2011). Unlike the trends over time for breast cancer, overall age-adjusted colorectal cancer incidence rates decreased consistently from 1995 to 2011. Colorectal cancer incidence rates were not consistently associated with higher income levels. A small increase of colorectal cancer incidence was observed in the lowest to third quintile and no increase of colorectal cancer incidence was observed from the third to the highest income quintile. When the results were stratified by tumor stage (last 2 columns in Table 2), unlike what was found for breast cancer in Table 1, tumor stage for colorectal cancer did not seem to modify the association between income and overall colorectal cancer incidence. In other words, both early and late stage colorectal cancer incidence rates slightly decreased with higher income.

**Table 2:** Number of men and women^a^ diagnosed with colorectal cancer, population estimates^b^, and age-adjusted colorectal cancer incidence rate^c^ in Texas, 1995–2011, stratified by median household income and tumor stage^d^ factors

Colorectal cancer: median household income quintiles	Counties	Cases^a^	Population^b^	Crude incidence rate per 100,000	Age-adjusted incidence rate per 100,000^c^	Early stage age-adjusted incidence rate per 100,000^c,d^	Late stage age-adjusted incidence rate per 100,000^c,d^
1995–1999							
Income Low < = $24,561	51	2,613	7,519,132	34.75	44.41	24.58	30.72
$24,561 < Income 2^nd^ < = $27,991	51	3,684	7,501,518	49.11	54.85	31.52	35.10
$27,991 < Income 3^rd^ < = $29,928	51	4,491	7,507,206	59.82	61.25	35.68	37.63
$29,928 < Income 4^th^ < = $33,652	51	10,311	20,848,852	49.46	60.50	30.93	32.53
$33,652 < Income High < = $68,003	50	21,738	55,378,453	39.25	61.48	29.92	32.49
Total 1995–1999	254	42,837	98,755,161	43.38	59.17	30.32	32.92
2000–2003							
Income Low < = $27,903	51	2,327	5,906,663	39.40	48.68	28.46	31.80
$27,903 < Income 2^nd^ < = $30,837	51	3,240	7,061,942	45.88	53.79	32.40	31.46
$30,837 < Income 3^rd^ < = $33,259	51	3,584	6,640,631	53.97	57.95	33.09	35.15
$33,259 < Income 4^th^ < = $37,476	51	5,695	9,934,760	57.32	61.86	35.11	31.87
$37,476 < Income High < = $76,188	50	22,503	56,441,381	39.87	59.17	29.49	30.29
Total 2000–2003	254	37,349	85,985,377	43.44	58.11	30.67	31.06
2004–2007							
Income Low < = $31,024	51	2,358	6,269,232	37.61	46.00	28.52	27.89
$31,024 < Income 2^nd^ < = $34,102	51	3,280	7,689,131	42.66	50.03	29.97	28.91
$34,102 < Income 3^rd^ < = $37,540	51	4,973	8,987,466	55.33	57.25	33.86	29.34
$37,540 < Income 4^th^ < = $42,068	51	4,971	9,094,178	54.66	56.86	31.52	29.81
$42,068 < Income High < = $75,467	50	22,220	60,318,599	36.84	51.78	25.67	26.77
Total 2004–2007	254	37,802	92,358,606	40.93	52.49	27.83	27.59
2008–2011							
Income Low < = $34,647	51	2,322	6,774,871	34.27	40.30	26.36	22.43
$34,647 < Income 2^nd^ < = $38,040	51	3,643	9,040,888	40.29	46.76	27.54	26.13
$38,040 < Income 3^rd^ < = $42,072	51	4,758	9,184,940	51.80	50.56	30.18	26.62
$42,072 < Income 4^th^ < = $48,438	51	10,432	26,337,018	39.61	46.86	24.43	24.12
$48,438 < Income High < = $80,876	50	16,391	48,659,170	33.69	44.73	22.87	22.60
Total 2008–2011	254	37,546	99,996,887	37.53	45.76	24.67	23.68

^a^Cases with unknown county were excluded

^b^Population estimates were obtained from US Census Bureau's Population Estimates Program

^c^Incidence rate was number of cases per 100,000 population, and was age adjusted to the 2000 US population

^d^Colorectal cancer early stage includes in situ and localized. Late stage includes regional and distant

**Fig. 2:**
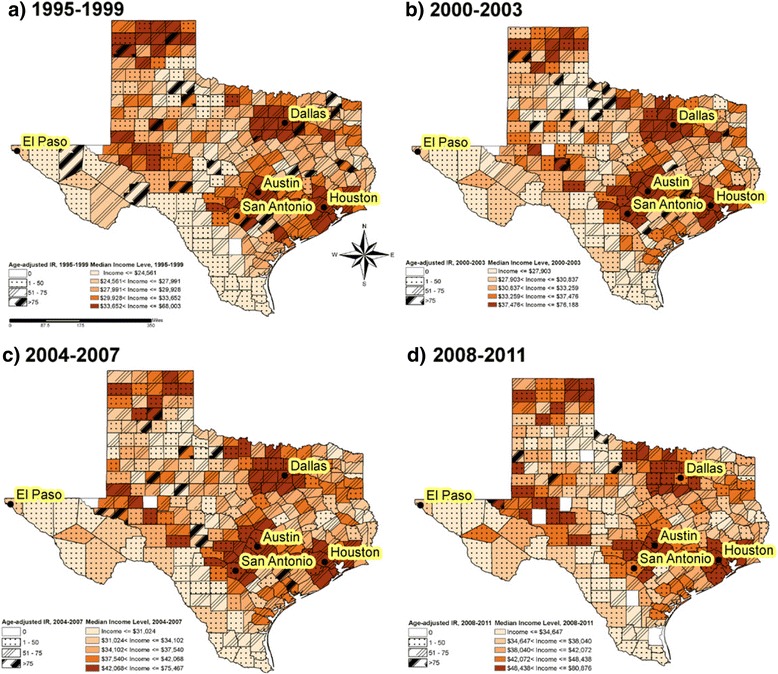
Geographic variations of colorectal cancer incidence adjusted for age and median household income in Texas, 1995–2011

Figure 2 provides the geographic distribution of age-adjusted colorectal cancer incidence rates associated with median income in Texas, stratified by four time period, (a) 1995–1999, (b) 2000–2003, (c) 2004–2007, and (d) 2008–2011. Although counties in the US-Mexico border area had lower age-adjusted colorectal cancer incidence rates, there was no clear pattern about the association between median income and colorectal cancer incidence rates. Spatial analysis detected significantly low cluster colorectal cancer incidence regions near US-Mexico border counties in the 1995–1999 and 2008–2011 periods (*P* < 0.001, Additional file 1: Figure S2).

### Breast and colorectal cancer incidence risk ratios

Table 3 presents the breast and colorectal cancer incidence rate ratios (IRR) by median household income quintiles for overall population and also stratified by urbanization (metro/urban/rural) using Poisson regression models that were adjusted for age, gender, race, and all possible two-way interactions. Compared to those in the lowest median income quintile counties in 2008–2011, overall age-adjusted breast cancer incidence rate was 69 % higher in counties with the highest income quintile (IRR = 1.69, 95 % CI: 1.56–1.82) and 22 % higher in counties with the 2nd lowest income quintile (IRR = 1.22, 95 % CI: 1.10–1.34). The association between breast cancer and incomes was stronger in metro and urban areas. For example, breast cancer incidence rate in metro area was 66 % significant higher in counties with the highest income quintile (IRR = 1.66, 95 % CI: 1.52–1.82) compared to the lowest median income quintile counties. In rural areas, breast cancer incidence rates appeared to be elevated with higher income quintile, but were not statistically significant with wide confidence intervals, partly due to small numbers. Breast cancer risk increased with increasing median income in all 4 time periods. On the contrary, colorectal cancer risk was not increased with increasing median income.

**Table 3:** Median household income specific breast cancer and colorectal cancer incidence risk ratios^a^, stratified by degree of urbanization, estimated by Poisson regression models in Texas, 1995–2011

Median household income quintiles	Incidence rate ratios (95 % CI)^b^			
Breast cancer	Overall	Metro	Urban	Rural
1995–1999				
Income 2^nd^	1.24 (1.13–1.36)	1.27 (1.13–1.43)	1.18 (1.01–1.39)	0.95 (0.61–1.50)
Income 3^rd^	1.45 (1.33–1.59)	1.58 (1.41–1.77)	1.35 (1.15–1.58)	0.88 (0.53–1.45)
Income 4^th^	1.35 (1.25–1.46)	1.39 (1.27–1.51)	1.39 (1.18–1.63)	0.92 (0.46–1.83)
Income 5^th^ (High)	1.55 (1.45–1.67)	1.63 (1.50–1.77)	1.09 (0.84–1.43)	1.60 (0.97–2.67)
2000–2003				
Income 2^nd^	1.41 (1.28–1.57)	1.55 (1.37–1.76)	1.21 (1.01–1.46)	0.80 (0.44–1.45)
Income 3^rd^	1.34 (1.21–1.49)	1.37 (1.18–1.58)	1.25 (1.05–1.48)	1.35 (0.78–2.34)
Income 4^th^	1.55 (1.41–1.71)	1.66 (1.48–1.86)	1.41 (1.17–1.71)	0.59 (0.27–1.29)
Income 5^th^ (High)	1.74 (1.60–1.89)	1.86 (1.68–2.06)	1.47 (1.15–1.88)	1.15 (0.62–2.16)
2004–2007				
Income 2^nd^	1.26 (1.14–1.40)	1.38 (1.22–1.56)	1.03 (0.86–1.24)	1.09 (0.57–2.12)
Income 3^rd^	1.42 (1.29–1.56)	1.61 (1.43–1.81)	1.11 (0.93–1.32)	0.93 (0.49–1.75)
Income 4^th^	1.38 (1.25–1.52)	1.46 (1.30–1.64)	1.24 (1.02–1.50)	0.57 (0.23–1.40)
Income High	1.67 (1.54–1.81)	1.80 (1.63–1.98)	1.19 (0.92–1.55)	0.66 (0.30–1.48)
2008–2011				
Income 2^nd^	1.22 (1.10–1.34)	1.19 (1.06–1.33)	1.35 (1.11–1.64)	0.94 (0.47–1.88)
Income 3^rd^	1.28 (1.16–1.41)	1.30 (1.16–1.46)	1.33 (1.10–1.61)	1.14 (0.61–2.16)
Income 4^th^	1.45 (1.34–1.58)	1.44 (1.31–1.58)	1.46 (1.18–1.82)	1.02 (0.45–2.33)
Income 5^th^ (High)	1.69 (1.56–1.82)	1.66 (1.52–1.82)	1.91 (1.47–2.48)	1.31 (0.66–2.62)
Colorectal cancer	Overall	Metro	Urban	Rural
1995–1999				
Income 2^nd^	1.40 (1.17–1.67)	1.27 (0.99–1.63)	1.29 (0.97–1.72)	0.54 (0.23–1.29)
Income 3^rd^	1.53 (1.28–1.82)	1.71 (1.35–2.16)	1.15 (0.85–1.54)	0.82 (0.31–2.17)
Income 4^th^	1.29 (1.10–1.50)	1.48 (1.23–1.78)	1.17 (0.87–1.58)	0.65 (0.17–2.55)
Income 5^th^ (High)	1.27 (1.10–1.46)	1.52 (1.28–1.81)	1.08 (0.66–1.78)	1.42 (0.55–3.68)
2000–2003				
Income 2^nd^	1.08 (0.90–1.30)	1.01 (0.79–1.29)	1.15 (0.84–1.58)	0.65 (0.24–1.74)
Income 3^rd^	1.33 (1.11–1.59)	1.37 (1.06–1.76)	1.14 (0.84–1.53)	0.71 (0.27–1.89)
Income 4^th^	1.36 (1.15–1.61)	1.54 (1.26–1.90)	1.14 (0.81–1.59)	0.64 (0.21–1.94)
Income 5^th^ (High)	1.14 (0.99–1.32)	1.32 (1.10–1.59)	1.11 (0.71–1.75)	0.42 (0.05–3.50)
2004–2007				
Income 2^nd^	1.21 (1.01–1.45)	1.08 (0.85–1.36)	1.16 (0.86–1.56)	1.25 (0.37–4.17)
Income 3^rd^	1.29 (1.09–1.53)	1.32 (1.06–1.63)	1.08 (0.80–1.45)	0.46 (0.12–1.73)
Income 4^th^	1.53 (1.30–1.81)	1.69 (1.38–2.06)	1.11 (0.81–1.54)	1.57 (0.39–6.36)
Income 5^th^ (High)	1.16 (1.00–1.34)	1.29 (1.08–1.53)	1.38 (0.90–2.10)	0.56 (0.06–5.59)
2008–2011				
Income 2^nd^	1.20 (1.01–1.43)	1.18 (0.94–1.48)	1.03 (0.76–1.39)	1.24 (0.43–3.56)
Income 3^rd^	1.51 (1.28–1.79)	1.70 (1.37–2.11)	1.02 (0.75–1.36)	0.93 (0.36–2.40)
Income 4^th^	1.30 (1.12–1.51)	1.53 (1.27–1.84)	1.05 (0.74–1.50)	3.13 (0.72–13.69)
Income 5^th^ (High)	1.21 (1.05–1.40)	1.44 (1.20–1.71)	1.09 (0.69–1.73)	0.85 (0.30–2.42)

^a^Poisson Regression model calculated incidence rate ratios (IRR) was adjusted for age, race/ethnicity, gender, median household income and all two-way interactions stratified by degree of urbanization/population

^b^ *IRR* incidence rate ratios was calculated by median household income quintiles and using first quintile-Median household income Low as reference group

Table 4 presents the risk of breast and colorectal cancer in association with median income and race/ethnicity by 4 time periods. Because of statistically significant interactions between median income and race/ethnicity, the association between cancer risk and median incomes was stratified by race/ethnicity. In non-Hispanic white women with breast cancer, the risk of breast cancer significantly increased with increasing median income in all time periods except in 2000–2003. However, in other ethnic women, breast cancer risk did not appear to increase with increasing median income. On the contrary, in men and women with colorectal cancer, we did not observe any pattern of increased risk of colorectal cancer with increasing median income in non-Hispanics whites. The risk of colorectal cancer appeared to decrease with increasing median income in racial/ethnic populations.

**Table 4:** Race and median household income specific breast cancer and colorectal cancer incidence risk ratios^a^, estimated by Poisson regression models in Texas, 1995–2011

Race/ethnicity	Incidence rate ratios (95 % CI)^b^			
Breast cancer	Income 2^nd^	Income 3^rd^	Income 4^th^	Income 5^th^ (High)
1995–1999				
Non-Hispanic Whites	1.01 (0.95–1.07)	1.07 (1.01–1.13)	1.10 (1.04–1.16)	1.24 (1.17–1.30)
Non-Hispanic Blacks	0.84 (0.65–1.10)	0.81 (0.63–1.05)	0.82 (0.64–1.04)	0.85 (0.67–1.07)
Hispanics	1.17 (1.09–1.25)	1.31 (1.20–1.42)	1.18 (1.11–1.26)	0.99 (0.93–1.05)
Others	0.44 (0.16–1.19)	0.99 (0.37–2.68)	0.53 (0.22–1.29)	0.41 (0.17–1.01)
2000–2003				
Non-Hispanic Whites	1.05 (0.98–1.12)	1.03 (0.96–1.10)	1.10 (1.03–1.17)	1.26 (1.19–1.34)
Non-Hispanic Blacks	0.75 (0.56–0.99)	0.68 (0.52–0.90)	0.67 (0.51–0.88)	0.70 (0.54–0.91)
Hispanics	1.11 (1.04–1.20)	1.23 (1.10–1.38)	1.33 (1.23–1.44)	1.10 (1.03–1.16)
Others	0.66 (0.29–1.51)	2.42 (1.12–5.22)	1.13 (0.53–2.38)	0.98 (0.49–1.97)
2004–2007				
Non-Hispanic Whites	1.01 (0.94–1.09)	1.07 (1.00–1.15)	1.07 (1.00–1.14)	1.27 (1.18–1.35)
Non-Hispanic Blacks	0.90 (0.68–1.19)	0.86 (0.66–1.11)	0.96 (0.73–1.24)	0.90 (0.70–1.16)
Hispanics	1.13 (1.05–1.21)	1.40 (1.27–1.54)	1.27 (1.17–1.37)	1.14 (1.08–1.21)
Others	0.94 (0.44–2.01)	1.22 (0.62–2.43)	1.64 (0.84–3.23)	1.06 (0.57–1.97)
2008–2011				
Non-Hispanic Whites	1.12 (1.05–1.21)	1.14 (1.07–1.22)	1.34 (1.26–1.43)	1.38 (1.29–1.47)
Non-Hispanic Blacks	0.88 (0.66–1.16)	0.86 (0.66–1.13)	0.95 (0.74–1.23)	0.93 (0.72–1.20)
Hispanics	1.12 (1.05–1.19)	1.22 (1.11–1.33)	1.13 (1.06–1.19)	1.14 (1.07–1.20)
Others	1.32 (0.77–2.26)	1.60 (0.95–2.69)	1.01 (0.63–1.63)	1.04 (0.65–1.67)
Colorectal cancer	Income 2^nd^	Income 3^rd^	Income 4^th^	Income 5^th^ (High)
1995–1999				
Non-Hispanic Whites	1.01 (0.94–1.10)	1.05 (1.01–1.10)	0.98 (0.92–1.06)	0.99 (0.93–1.06)
Non-Hispanic Blacks	0.89 (0.69–1.15)	0.82 (0.64–1.05)	0.65 (0.51–0.81)	0.64 (0.51–0.80)
Hispanics	1.28 (1.17–1.40)	1.74 (1.56–1.93)	1.37 (1.27–1.48)	1.14 (1.06–1.23)
Others	0.22 (0.08–0.65)	0.51 (0.13–2.08)	0.20 (0.09–0.47)	0.12 (0.06–0.28)
2000–2003				
Non-Hispanic Whites	0.95 (0.88–1.03)	0.94 (0.87–1.02)	0.95 (0.88–1.03)	0.92 (0.85–0.98)
Non-Hispanic Blacks	0.63 (0.47–0.82)	0.56 (0.43–0.73)	0.50 (0.38–0.64)	0.46 (0.36–0.59)
Hispanics	1.06 (0.97–1.15)	1.53 (1.34–1.74)	1.51 (1.37–1.65)	1.04 (0.97–1.11)
Others	0.28 (0.10–1.77)	0.84 (0.30–2.35)	0.49 (0.19–1.26)	0.14 (0.06–0.35)
2004–2007				
Non-Hispanic Whites	0.95 (0.87–1.04)	0.91 (0.84–0.98)	0.95 (0.88–1.03)	0.86 (0.79–0.92)
Non-Hispanic Blacks	0.62 (0.47–0.81)	0.58 (0.45–0.74)	0.55 (0.43–0.71)	0.49 (0.39–0.62)
Hispanics	1.09 (1.01–1.18)	1.75 (1.59–1.94)	1.43 (1.31–1.56)	0.99 (0.93–1.06)
Others	0.29 (0.12–0.71)	0.44 (0.19–0.99)	0.59 (0.26–1.36)	0.13 (0.06–0.27)
2008–2011				
Non-Hispanic Whites	1.04 (0.96–1.14)	0.98 (0.90–1.06)	0.93 (0.87–1.01)	0.88 (0.82–0.95)
Non-Hispanic Blacks	0.69 (0.52–0.91)	0.64 (0.49–0.83)	0.57 (0.44–0.74)	0.52 (0.40–0.67)
Hispanics	1.06 (0.99–1.15)	1.54 (1.40–1.70)	1.05 (0.98–1.12)	0.95 (0.89–1.02)
Others	1.31 (0.52–3.27)	2.17 (0.92–5.10)	0.57 (0.25–1.30)	0.53 (0.24–1.18)

^a^Poisson Regression model calculated incidence rate ratios (IRR) was adjusted for age, race/ethnicity, gender, median household income and all two-way interactions

^b^ *IRR* incidence rate ratios was calculated by median household income quintiles and using first quintile-Median household income Low as reference group

## Discussion

This study demonstrated that breast cancer risk increased with increasing median income, whereas colorectal cancer risk slightly decreased with increasing median income. In addition, the study examined the risks of breast and colorectal cancer risk by race/ethnicity and degree of urbanization and highlighted the spatial variations in Texas where the breast and colorectal risks were lower in particular regions as compared to other geographic areas. This appears to be the first study in Texas to assess the relationship between median household income and age-adjusted breast and colorectal cancer incidence rates.

The findings of this study were consistent with those of other studies outside Texas. Numerous studies had previously shown that breast cancer risk was associated with higher income and colorectal cancer risk was associated with lower income in the U.S [5–13]. For example, Clegg and colleagues studied the risk of cancer in association with SES in the 11 SEER areas in 1973–2001 and found that age-adjusted breast cancer incidence increased from 136.35 cases (per 100,000 population) in those with family income of < $12,500 per year to 158.15 cases in those with family income of ≥ $50,000, whereas the age-adjusted colorectal cancer incidence (in both men and women combined) decreased from 69.55 to 64.09 [11]. Klassen and Smith reviewed 90 studies from around the world that were published between 1978 and 2009 on breast cancer and social class, concluding that breast cancer incidence continued to be higher in high social class populations than in low social class populations [46]. On the contrary, Aarts and colleagues reviewed 62 studies published between 1995 and 2009 on colorectal cancer incidence and socioeconomic status and concluded that a lower SES was associated with higher colorectal cancer incidence in the U.S. and Canada, although the findings on the SES and colorectal cancer risk in Europe were different [26]. Also, the gap in colorectal cancer incidence between high and low socioeconomic status was narrowing over time [47, 48].

The relationships between cancer risks and socioeconomic status are complex, especially so for breast cancer. Multiple reasons and explanations have been discussed previously, including social class as a marker for biological and behavioral differences, differential access to medical facilities, different health awareness for disease screening or early detection, and different exposures to environmental pollution particularly in metro and urban areas, lifestyle, stress, and work factors [49–54]. High social class and income have been documented to influence mothers and daughters in their reproductive life and related factors, for example, earlier onset of menarche, delayed age for first birth and menopause, fewer number of children, and perhaps more use of hormone replacement therapies, all of which were associated with a prolonged exposure to hormones and an increased risk of breast cancer [55–58]. These hormone-related factors may be one of the reasons why there was no such an association between social class and increased risk of colorectal cancer because colorectal cancer is not a hormone-associated tumor. Furthermore, when mammogram as an effective screening tool was implemented, breast cancer incidence increased sharply due to screening. For example, breast cancer incidence in the U.S. was increased from 112 cases per 100,000 women in the early 1980s to 234 cases per 100,000 women in the late 1990s [59]. This increase was particularly evident in women with higher income, better health insurance coverage, and greater access to screening facilities. For this reason, we stratified the results by tumor stage and found that higher income was significantly associated with an increased early stage breast cancer incidence in 4 different time periods. On the contrary, no sharp increase in colorectal cancer incidence was observed after cancer screening in the U.S [2, 60, 61]. In this study we also found that early stage colorectal cancer incidence was not associated with income and time periods. Moreover, genetic and environmental factors were associated with an increased risk of breast and colorectal factors. For example, a diet that is high in red meats or processed meats has been well documented to increase cancer risk overall and colorectal cancer risk in particular [62–65]. This type of diet was associated with lower income, which was consistent with what we found in this study on the higher risk of colorectal cancer in those men and women with lower income [66, 67]. Finally, we observed significant clustered counties for low breast cancer incidence in southwest US-Mexico border in all four time periods. This border area consisted of low income counties and a majority of people in these counties were Hispanics with Mexican origin. The finding of a low breast cancer incidence was consistent with previous studies [6–9, 11, 46]. It was reported that breast cancer incidence rate in Hispanic women was 26 % lower than in non-Hispanic white women and these risk differences were likely attributed to differences in some potential risk factors for breast cancer such as lower age at birth and more children in Hispanic women [68]. However, we also observed significant clustered counties for low colorectal cancer incidence in this border area in the 1995–1999 and 2008–2011 periods but not in the 2000–2007 period. Although numerous studies concluded an association between low income and high colorectal cancer incidence, the low colorectal cancer incidence in these US-Mexico border counties might be partially explained by the fact that those Hispanics with Mexican origin had a lower risk than those with other Hispanic origins in Puerto Rico, South and Central America [68].

Because the above specific risk factors (such as number of children) and screening patterns for breast and colorectal cancer were not measured in our study, this report cannot address how these factors might explain the observed associations between income and cancer risk, but only demonstrated that higher income was associated with an increased risk of breast cancer and a lower risk of colorectal cancer in Texas. In particular, the mechanism of the finding that the trend of an increased risk of breast cancer in association with higher median income was statistically significant only in non-Hispanic white women after adjusting for age may need further research. There were some other limitations to be noted in this study. First, the income variable was the median household income at county level but not at individual level. Hence, there was a potential ecological fallacy in which the income at group level may not necessarily represent the income at individual level. Second, median household income alone may not be a good proxy for socioeconomic status. Ideally, education, occupation, and health knowledge, which are strongly associated with higher cancer screening rates and healthy lifestyles, should all be taken into consideration in the analyses. Third, the denominator populations by county in the non-census years were estimated by the U.S. Bureau of Census. We were unable to verify specific populations by year and age, particularly when Texas has one of the largest population growth and change in demographics in the U.S. The lack of accuracy in population estimates might have led to biased calculations for cancer incidence rates.

## Conclusion

In conclusion, this statewide and population-based study demonstrated that higher income was associated with an increased risk of breast cancer and a decreased risk of colorectal cancer in Texas. There were also geographic variations with cancer cases clustered in high and low risk areas in Texas. Future studies may need to explore more factors that might explain these income and cancer risk associations and geographic variations.

### Ethics approval and consent to participate

The Institutional Review Board of the Texas Department of State Health Services and the Committee for the Protection of Human Subjects at the University of Texas Health Science Center granted ethics approval to our study. The informed consent was waived because the study was retrospective in design and from public datasets.

### Consent for publication

Not applicable because this manuscript does not contain any individual persons data.

### Availability of data and materials

The de-identified datasets on incident breast and colorectal cancer cases in this study cannot be shared due to the release policy requirement by the Texas Cancer Registry (TCR). However, these public-use datasets can be obtained from TCR by following a few appropriate procedures at http://www.cprit.state.tx.us/texas-cancer-registry [22]. Information on the county population estimates, median household income, and population age groups at the county level was obtainable from the U.S. Census Bureau without needing approval at http://www.census.gov/did/www/saipe/index.html [23, 29].

## Additional file

Additional file 1: Supplemental Statistical Methods and Figures. (DOCX 604 kb)

## Abbreviations

ESRI: Environmental Systems Research Institute
GIS: geographic information system
ICD-O: International Classification of Diseases-Oncology
IRR: incidence rates ratio
NOS: non otherwise specified
RUCC: urban/rural continuum codes
SAS: statistical analysis system
SEER: surveillance, epidemiology, and end results
SES: socioeconomic status
TCR: Texas Cancer Registry
U.S.: United States
USDA: U.S. Department of Agriculture
