# Supplementary material for: Risks of developing breast and colorectal cancer in association with incomes and geographic locations in Texas: a retrospective cohort study
Source: BMC Cancer. 2016 Apr 26;16:294. doi: 10.1186/s12885-016-2324-z (PMC4847204; doi:10.1186/s12885-016-2324-z)

**Supplemental Materials**

**Statistical analyses:**

Getis-Ord G spatial statistics is used to reveal local clustering of specific phenomena.^36^ G statistics is defined as:

$$G=\frac{\sum_{i=1}^{n} \sum_{j=1}^{n} w_{i,j}x_{i}x_{j}}{\sum_{i=1}^{n} \sum_{j=1}^{n} x_{i}x_{j}}$$

Where $w_{i,j}$ is the spatial weight between i^th^ and j^th^ county. $x_{i}$ is the i^th^ county cancer incidence rate and $x_{j}$ is the j^th^ county cancer incidence rate. A positive and significant z-value for a G statistics indicates spatial clustering of high incidence rates of hot spot areas, whereas a negative and significant z-value indicates spatial clustering of low incidence rates of cold spot areas.

We calculated G statistics separately for each study time period and then used geographic information system (GIS) maps to identify cluster regions with high and low cancer incidence rates after adjusting for age and median household income.

The Poisson regression model adjust with covariates takes the following form:

$$y_{i}\sim Poisson(\lambda_{i})$$

$$\log\left( \lambda_{i} \right)=\alpha+\beta_{1}\left( \mathrm{Income} \right)+\beta_{2}\left( \mathrm{Age} \right)+\beta_{3}\left( \mathrm{Gender} \right)++\beta_{4}\left( \mathrm{Race} \right)+\beta_{5}\left( Income*Age \right)+\beta_{6}\left( Income*Gender \right)+\beta_{7}\left( Income*Race \right)+\beta_{8}\left( Age*Gender \right)+\beta_{9}\left( Age*Race \right)+\beta_{10}\left( Gender*Race \right)+\log\left( n_{i} \right)$$

Where$i=1,\ldots, 254,$; $y_{i}$ is the number of cancer cases in i^th^ county; expected count of $y_{i}$ is $E\left( Y \right)=\lambda_{i}$, $log(n)$ is referred to as an offset variable; n is the number of total population in the i^th^ county;$\alpha$ is the intercept; $\beta_{1}, \ldots, \beta_{10}$ $\beta_{1}, \ldots, \beta_{9}$are the coefficient estimates for fix effects.

**Figure S1: Cluster analysis results of female breast cancer incidence rates, adjusted by age and median household income in Texas, 1995-2011.**


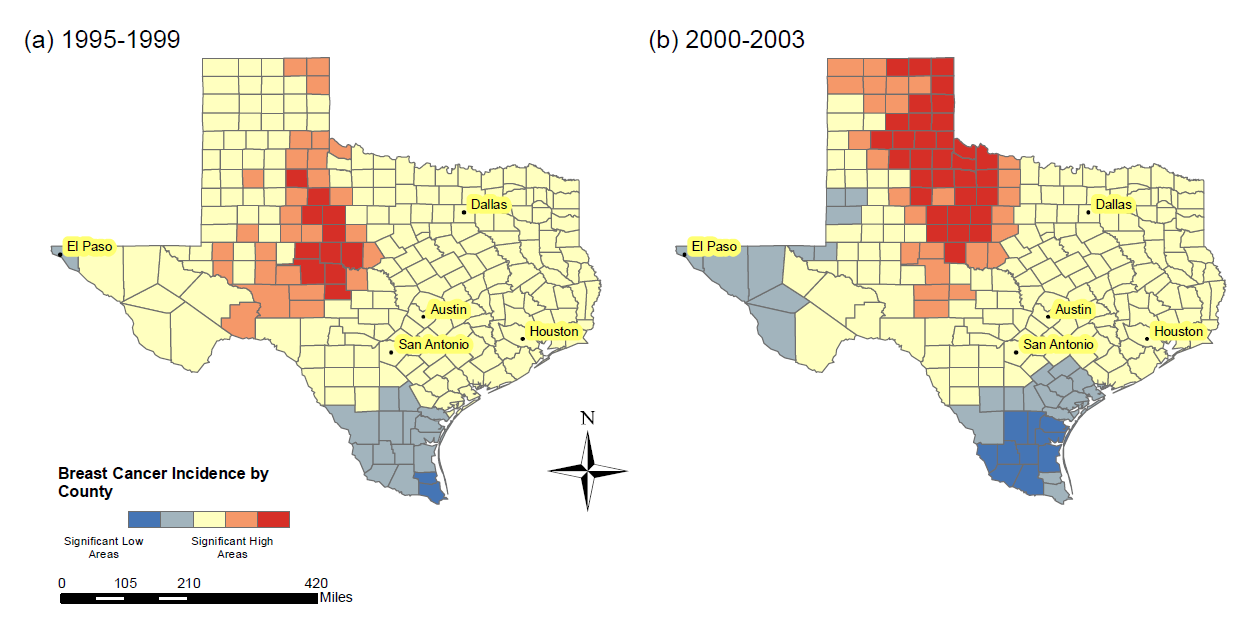


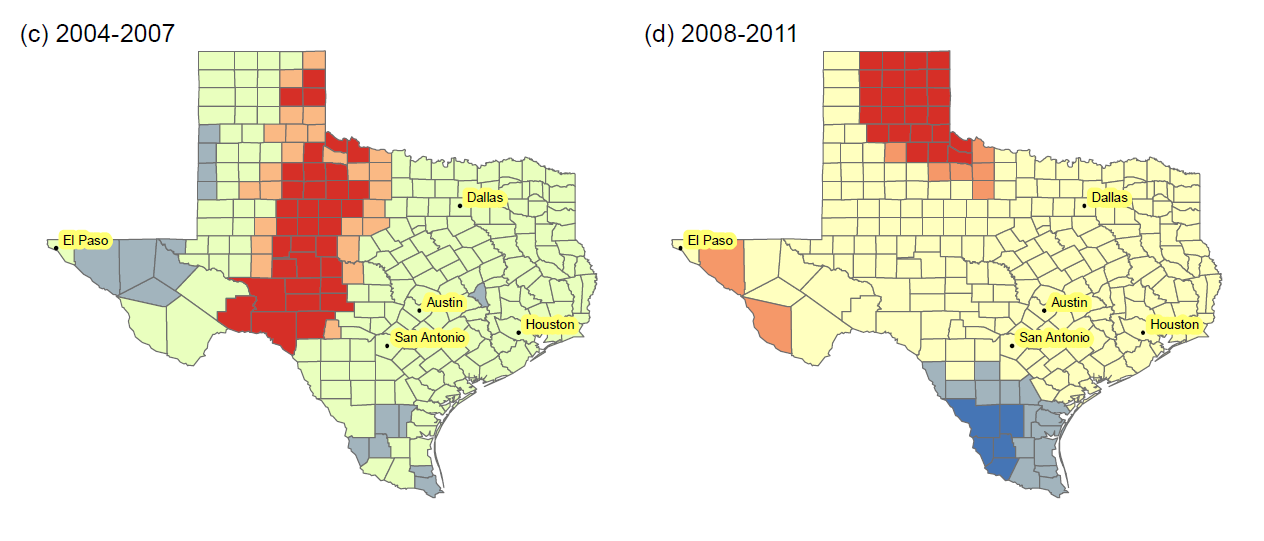


**Figure S2: Cluster analysis results of colorectal cancer incidence rates adjusted by age and median household income in Texas, 1995-2011.**


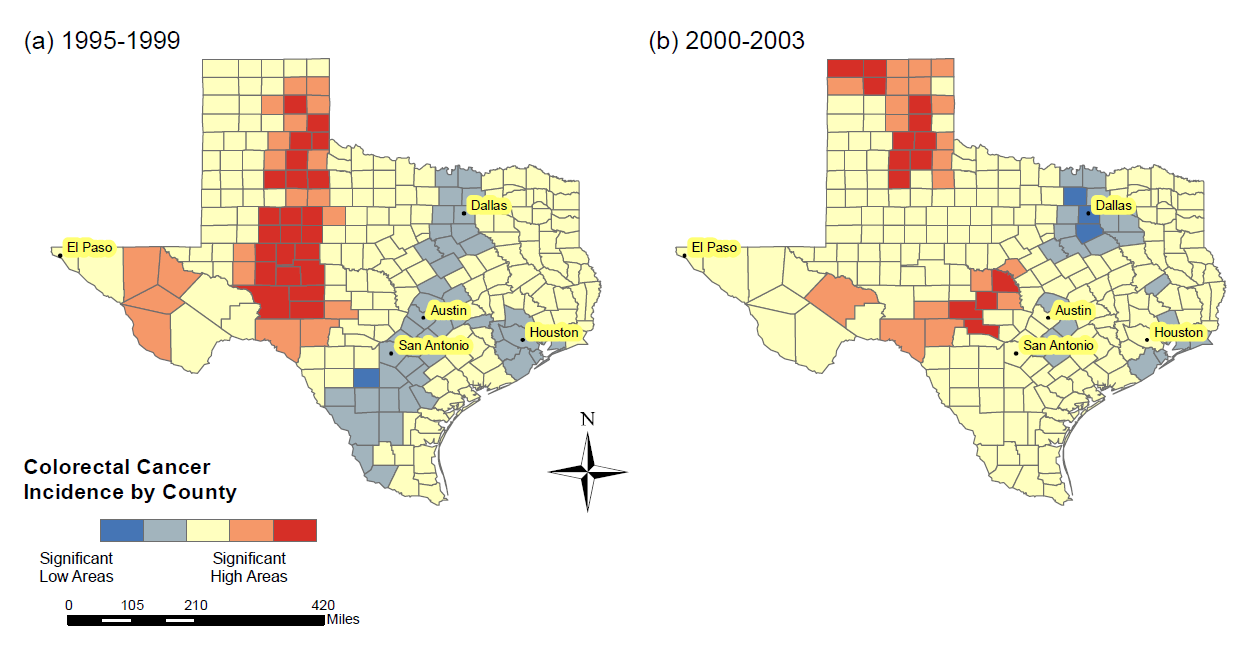


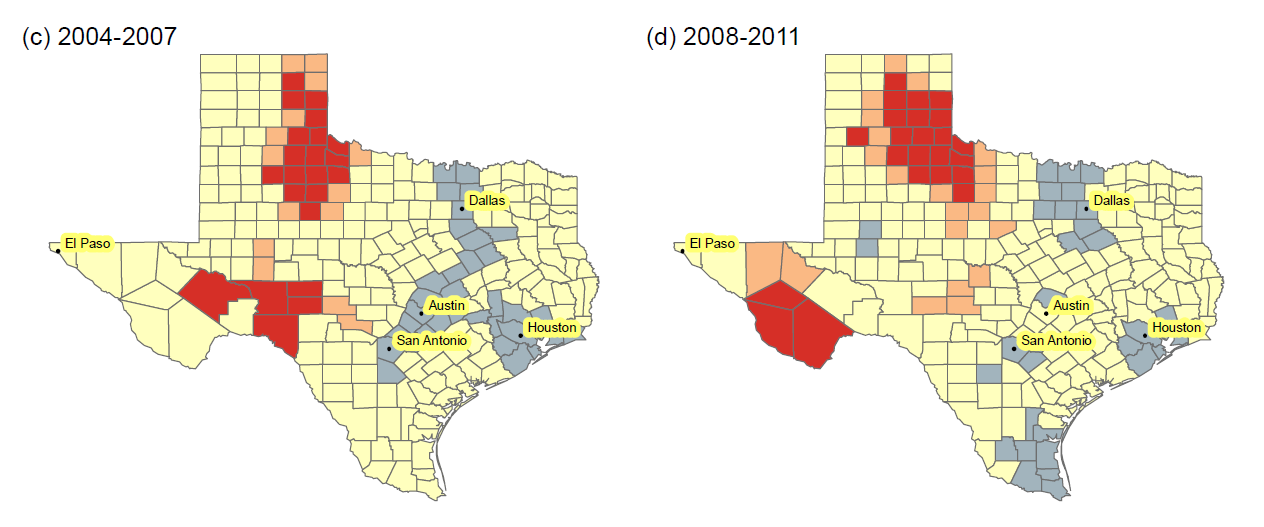

Supplement: Additional file 1: — Supplemental Statistical Methods and Figures. (DOCX 604 kb) [file 12885_2016_2324_MOESM1_ESM.docx]
